# Our French Visitors

**Published:** 1904-10-22

**Authors:** 


### Our French Visitors.

The visit of French physicians and surgeons to London, and the reception which awaited them at the hands of their English *confrères*, are likely to form by no means an insignificant link in that chain of friendly relations by which the two peoples are becoming more and more closely united; and it will, we hope, be the precursor of other visits of a similar kind. As originally planned, it was mainly intended to afford, to the younger members of the profession in Paris, and especially to the classes analogous to the house physicians and house surgeons of our own hospitals, an opportunity of seeing English medical institutions; and it was not until its success was manifestly assured that a desire to take part in it was evinced by any considerable proportion of their senior colleagues. By that time, unfortunately, the expedition was numerically complete, and no further enlargement of it was thought compatible with the full attainment of the ends for which it had been promoted. We may reasonably hope that on some near future occasion the visit will be repeated on a somewhat different plan, and for the express purpose of offering English hospitality to the most distinguished of the French faculty, who would, we are sure, be cordially welcomed both in our hospitals and in the homes of those by whom the hospitals are officered, and to whom they are indebted for whatever celebrity they may have gained. Fifty years ago it was extremely common, more common than it has been recently, for English medical students to complete their education in Paris, and the custom laid the foundation of many abiding friendships between men who had first met as students, and who had afterwards risen to eminence in their respective countries. Although no publicity was given to the visits, it was then by no means unusual to meet the most distinguished French physicians and surgeons in London medical houses. The single fact of the degree in which the discoveries of Pasteur suggested the improvement of surgery to Lord Lister affords a sufficient proof, if proof were wanted, of what may be called the natural intellectual fertility of an Anglo-French alliance; and, just as such an alliance would be almost omnipotent in the domains of war and of politics, so it would be the source of incalculable advantages in the domain of science. The circumstances of the two countries, and the conditions of living of their respective populations, are just sufficiently different to insure complete trials, and hence exhaustive experience, of the merits or defects of methods which were transplanted from one to the other. In bygone times it was somewhat of a reproach to French surgery that the disease or the operation appeared to receive more consideration than the patient, and it would no doubt have been said, by those against whom such an accusation was directed, that our consideration for the patient sometimes placed artificial difficulties in the way of affording him relief. No doubt each nation may learn much from the other; and a natural consequence of frequent and friendly intercourse will be the removal of any prejudice on either side. Both alike will be more disposed than formerly to obey the apostolic injunction, to prove all things, and to hold fast that which is good.

We are the more gratified at the visit of our French colleagues, and at the prospects which it seems to hold forth, because we think there has been a manifest tendency in this country of late years somewhat to neglect or to undervalue French medical science, and in a corresponding degree to overestimate that which has been "made in Germany," and which, as it seems to us, has often been of a very inferior character. The exaggerated importance attached to some varieties of German work by many of our countrymen has mainly originated, in all probability, in quarters outside the medical profession; but there can be no doubt that, partly because these were influential, and partly for want of precise examination into the actual facts, it has penetrated too deeply into the profession itself. The prevailing absurd and utterly unfounded exaggerations of the value of German ophthalmology may be taken as an instance very much in point, and these have been kept afloat, so to speak, not only by his Highness "the public," but even by practitioners who could not have described a single gain in this department for which the world has been indebted to any German, and who remained in contented ignorance of the work, or even of the names, of such men as Abadie and Darier. We should be sorry to do any injustice to the German school, but there seems only too much reason to believe that the tortuosity of the national politics is not wholly without representation in the national science, and that philosophers with one eye on the work of the laboratory have not been superior to the business of keeping the other steadily fixed upon commercial relations, upon the requirements of authority, or upon the main chance. The world has seen no more majestic example of devotion to science, of the love of truth for its own sake, of the free diffusion of inestimable knowledge, of the rejection of everything which was meretricious, than that which was afforded by Pasteur, for whom, except perhaps in our own Newton, it would be difficult to find a parallel in history. It must be assumed, we think, that the spirit of this great man survives in his compatriots, and that the influence of his mind and of his character will be deeply engraven upon the French research of generations yet to come. The work of the great Institute which bears his name is likely to furnish guidance to many who will follow in his footsteps, and who are not likely to be influenced in their declarations, either by the fluctuations of the market or by the desires of persons in authority. The France of to-day is manifestly shaking herself free of many hindrances which restrained her development under the second Empire, and is more and more showing her capacity to assume a place of light and leading among nations. In the development of her future the advancement of the medical sciences will hold no insignificant position, and we gladly hold out a hand of fellowship to those by whom this advancement is being accomplished.

